# Liver Flukes Egg Infection and Associated Risk Factors in White Fulani Cattle Slaughtered in Wukari, Southern Taraba State, Nigeria

**DOI:** 10.1155/2019/2671620

**Published:** 2019-03-19

**Authors:** P. A. Shinggu, O. T. Olufemi, J. A. Nwuku, E. B. T. Baba-Onoja, P. D. Iyawa

**Affiliations:** ^1^Department of Animal Production and Health, Faculty of Agriculture and Life Sciences, Federal University Wukari, Taraba State, Nigeria; ^2^Department of Veterinary Public Health and Preventive Medicine, Faculty of Veterinary Medicine, University of Jos, Plateau State, Nigeria

## Abstract

*Fasciola, Fascioloides*, and* Dicrocoelium* cause liver fluke diseases in ruminants and are of zoonotic and economic importance. This cross-sectional study aimed to determine the prevalence of liver fluke egg infection in White Fulani Cattle slaughtered in Wukari Cattle market abattoir in Wukari, Taraba State. A total of 262 gallbladders were collected and their contents were analyzed for the presence of eggs of liver flukes using sedimentation technique. Descriptive analysis was done using SPSS version 20 for window and Pearson's Chi-Square (*χ*^2^) was used to evaluate the association between variables.* Fasciola* and* Dicrocoelium* eggs were encountered. Only 74 (28.2%) samples were positive for* Fasciola* egg and 211 (80.5%) for* Dicrocoelium*. The prevalence of liver fluke infection revealed 25% and 28.6%* Fasciola* eggs for male and female animals, respectively, while lancet fluke had 83.3% for male and 80.3% for female.* Fasciola* eggs were recovered in 20.8% of animals aged less 3 years (<3yrs) and 29.9% was recorded in animals 3 years and above (≥ 3yrs) while 81.3% for animals ≥ 3yrs and 77.1% for animals <3yrs were recorded for* Dicrocoelium *eggs. The body condition score-based prevalence for* Fasciola* yielded 38.1%, 26.8%, and 14% for poor, average, and good, respectively, while* Dicrocoelium* yielded 85.7%, 79.3%, and 85.7%. There was a significant difference between the body condition scores for fasciolosis. Only 59% harboured single infection with eggs of* Dicrocoelium* (P< 0.05) while 6.9% harboured* Fasciola *eggs. Mixed infection associating* Fasciola* and* Dicrocoelium* was observed in 21.4% of the sample cattle. Liver fluke infections: fasciolosis and dicrocoeliosis occur among White Fulani cattle in Wukari and these infections are associated with the body condition score of the animals. This greatly affects the cattle production. There is a need to institute adequate control programmes complemented with good well-planned management practices in any production system involving cattle in Wukari.

## 1. Introduction

Liver fluke infections of cattle are caused by digenean trematodes of the genera* Fasciola* and* Dicrocoelium* widely referred to as common liver fluke and lancet fluke of ruminants, respectively. Diseases caused by these two genera are fasciolosis and dicrocoeliosis with etiologic agents in tropical Africa as* Fasciola gigantica* and* Dicrocoelium hospes*, respectively [1]. The lifecycle of these trematodes involves mollusc as an intermediate host [2] and the tropical environment in association with the relative abundance of a snail intermediate host that propagates the sporocyst, redia, and cercarial stages of the parasites [3]. Cattle usually get infected after ingesting the metacercarial stage while grazing or in drinking water for* Fasciola* and following the ingestion of infected ant for* Dicrocoelium* [2]. These parasites migrate to the bile ducts causing severe pathological changes in the liver. The disease usually results in decreased production of meat, milk, and wool, secondary bacterial infections, fertility problems, loss of weight, poor carcass quality, and great expenses on anthelmintics medication [4, 5]. In addition to their veterinary importance, these flukes are also known zoonosis affecting a number of the human population [6, 7].

Several researchers at different times in various places have documented the prevalence and economic importance of liver fluke [8–11]. Findings from the northeast subregion of Nigeria, by Nwosu and Srivastava (1993) in Maiduguri, reported a prevalence of 21.1 and 18.3% for* Fasciola *and* Dicrocoelium*, respectively, while Ardo et al. (2013) in Yola documented a prevalence of 22.08% for Fasciola.

Taraba state occupies the southern part of northeastern Nigeria. It is a unique state with three distinct natural vegetation belts comprising the Sahel Savannah in the northern part, the Guinea Savannah in the central and southern regions, and an almost temperate region (weather) on the Mambilla Plateau (Tarabastate.gov.ng 2016). The state also has three major rivers, namely, River Benue, River Taraba, and River Donga, where the people along the river routes engage in extensive agricultural activities. Cattle, sheep, and goats are reared in large numbers, especially in the southern part the state and the Mambilla Plateau. The state is one of the leading contributors of livestock and livestock products to the Nigerian livestock industries. Wukari is a medium sized town positioned in the southern part of the state which falls within the southern guinea savannah ecological zone bordered by these major rivers. The climate and vegetation of this area contrast the arid and semi-arid vegetation that characterizes the wider northeastern region. Despite the importance of this area as one of the major contributors to the livestock industry, there is a paucity of information on parasitic diseases of livestock in this area. It thus becomes appropriate to evaluate the prevalence of liver fluke infection in White Fulani cattle slaughtered in Wukari abattoir. Information obtained from the study will give a more complete picture of the status of the disease in southern Taraba State.

## 2. Materials and Methods

### 2.1. Study Area

This study was carried out in Wukari, a medium size town located within Latitude 07^0^.85′ N and longitude 09^0^.78′ E of the equator (World Atlas 2015). The town is within the Southern Guinea savannah ecological zone. River Donga flows through the area and the Benue River forms a boundary with Plateau and Nasarawa state to the west. This area has an average rainfall of 1205 mm (Climate-data.org.). The rainy season lasts for 7 months (April to October) and is followed by a short dry season from November to March [12]. Cattle slaughtered at the abattoir are representatives of the various herds in Wukari and neighbouring local government areas in southern Taraba state

### 2.2. Animal Selection and Sample Collection

Study animals were randomly selected each day from the slaughter slabs of Wukari abattoir. A total of 262 cattle were sampled. The breed, clinical status, age, and sex of each animal were determined and recorded. All cattle sampled were categorised into two age groups: <3 years and ≥3 years, based on the number of grazing seasons experienced. The body condition score for each cattle was estimated ranging from Score 1 (poor), Score 2 (average), to Score 3 (good) [13].

After slaughter and evisceration, intact gallbladder of each of the selected animals was carefully collected into properly labelled clean polythene bags and transported on ice to the Biology Laboratory, Federal University Wukari, Nigeria, for recovery and identification of eggs of liver fluke.

### 2.3. Laboratory Sample Analysis

Each gallbladder was cut open and the entire bile routinely washed with tap water into a 500 ml beaker. The bile content was then sieved through a tea strainer and the filtrate allowed to settle for 2 hours; the supernatant decanted leaving the sediments. The sediments were pipetted and drops were placed on a clean glass slide and a coverslip applied before examination under the microscope at X10 objective lens. Where it was anticipated that the bile could not be examined over a 24-hour period, drops of formaldehyde were added prior to the time they will be viewed. Identification of eggs of these flukes was based on standard morphological characteristics and size of eggs as described in [3, 14, 15].

### 2.4. Data Analyses

Data obtained were subjected to statistical analysis using the SPSS version 20.0 for windows. The difference among variables was evaluated by Pearson's Chi-Square Test and values of P≤0.05 were considered statistically significant.

## 3. Results

Out of the 262 bile specimens examined, 74 (28.2%) samples were found positive for* Fasciola* egg and 211 (80.5%) for* Dicrocoelium *spp. The distribution of liver fluke parasites in slaughtered cattle by sex, age, and body condition score is presented in Table 1. The prevalence of liver fluke infection between the two sexes in the current study revealed 25 and 28.6%* Fasciola* prevalence for male and female, respectively, while lancet fluke recorded a prevalence of 83.3% for male and 80.3% for female. There was no statistical difference between the two sexes (P >0.05).* Fasciola* infection in the animals ≥3 years was observed to be higher (29.9%) as compared to the animals < 3years: 20.8%. This study also identified that female animals were the most slaughtered at the Wukari abattoir; they account for 90.8% of the total cattle slaughtered. A similar high prevalence of 81.3% for the animals ≥3 years as against 77.1% in animals < 3 years was recorded for* Dicrocoelium*. Chi-square analysis showed the variation observed for the animals ≥3 years and < 3 years was also not statistically significant (P > 0.05).

On the basis of body condition of the sampled animals, more cases of fasciolosis were recorded in the poor body conditioned category (38.1%) as compared to those identified as average (26.8%) and good (14.3%) body conditioned animals. The variation for* Fasciola* in the poor body condition group was significantly higher (P< 0.05) than the average and good body conditioned grades. However, there was no significant difference for* Dicrocoelium* (P >0.05) among the three body condition scores with the prevalence of 85.7%, 79.3%, and 85.7% for poor, average, and good body condition scores, respectively.

The prevalence of single and mixed infections established in this study is shown in Table 2. Significantly large proportion 155 (59%) of the sampled animals were identified to harbour a single infection with eggs of* Dicrocoelium*. This prevalence significantly (P< 0.05) varied from 18 (6.9%) recorded for* Fasciola*. Mixed infection associating* Fasciola* and* Dicrocoelium* was observed in 56 (21.4%) of the sample cattle.

## 4. Discussion

The result of this study indicates that the White Fulani breed of cattle slaughtered in Wukari is naturally infected with two varieties of liver flukes. The two species encountered were* Fasciola* and* Dicrocoelium* with a prevalence of 28.2 and 80.5%, respectively. They are pathogenic to ruminants and also among important zoonotic trematodes of public health importance [7, 16]. These species of liver flukes were earlier reported from studies conducted in cattle in other parts of Nigeria [8, 10, 17]. The prevalence of* Fasciola* and* Dicrocoelium* infections observed in this present study was however high as compared to the 1.7% for* Fasciola* and 35.4% for* Dicrocoelium* reported by Ulayi et al., 2007, in Zaria, north-central Nigeria, and 21.1%* Fasciola* and 18.3% for* Dicrocoelium* documented by Nwosu and Srivastava (1993) in Maiduguri, northeast of the country. The disparity in findings, as the studies were conducted in the same northern region of the country, does suggest that the prevalence of liver fluke infections varies with differences in geographical locations. The climatic elements and the ecological features of southern guinea savannah, the present study area, contrast those of the arid and semiarid region that characterized the wider north and northeast subregion of Nigeria. These elements exert a major influence on the development and survival of both the potential intermediate hosts and the developmental stages of these flukes. In addition, the pastoral system of management where herds move together to different places in search of pasture particularly along the plains of the three major rivers (Taraba, Benue, and Donga) which runs in the southern part of Taraba state does favour the transmission of these parasites.

Majority of the animals sampled were female (90.8%). This is probably because female animals form bulk of the herds in the area as they are mostly retained for breeding purposes and milk production. They are however normally culled off only when they have stopped bearing off-springs and have attained old age. The male counterparts, on the other hand, are normally disposed of immediately after attaining adult size with only a few being left for breeding purposes. The lack of significant variation (P> 0.05) in the prevalence of liver fluke between the male and female groups showed that male and female present the same chance of being infected if they are exposed to the same management system. This finding contradicts the reports of Adedokun et al. (2008) and Ulayi et al. (2007) who reported higher incidence in females than in males and was attributed to host intrinsic factor and longevity of the female in the herds; however this study confirmed the reports of Magaji et al. (2014) and Ieren et al. (2016) who also reported lack of significant variation between male and female cattle.

In the animals < 3 years and ≥ 3 years, respectively, there was no significant difference in the infection rates (P> 0.05) among different age groups. A similar observation was recorded by Tsegaye et al. (2012); this could be attributed to the system of management practised where animals are not segregated based on age. This system of management often exposes animals of all ages to equal risk of acquiring liver fluke infection. However, the current findings contradict Vassilev (1999) who reported an increasing prevalence of* Fasciola* in cattle as age increases which was attributed to longer exposure time and accumulation of fluke in the liver compared to the animals aged < 3 years. On the contrary, results indicating an inverse correlation of prevalence rate and age of cattle were reported by Getu et al. (2015) and this was associated with acquired immunity following several exposures to liver fluke infections [18].

The result of the present study indicates that body condition of animal has a significant association with the occurrence of* Fasciola* infection. The prevalence was significantly higher (P<0.05) in poor body conditioned animals than of average and good body condition groups. Similar observations were made by Getu et al. (2015) and Negesse and Mohammed (2014). Poor body conditioned animals resulting from nutritional or other health challenges are often associated with low resistance to parasitic infections; such animals when infected with liver flukes often end up with an exacerbated condition. This is because helminths infected animals give priority to the reversal of the pathophysiological consequences of parasitism over other body functions and growth [19]. Nutrients are thus not effectively utilized for body maintenance and growth. This results in cachexia, particularly at the chronic stage of bovine fasciolosis. However, despite the fact that* Dicrocoelium* recorded high prevalence, its distribution among the three body conditions had no significant difference (P>0.05). This observation could be attributed to the mild pathology caused by lancet fluke. This fluke migrates directly up the biliary duct system of the liver without penetrating the liver parenchyma [16, 20]. This migration in cattle causes less pathology and produces mild signs as compared to* Fasciola* infection.

Despite its widespread presence among grazing animals* Dicrocoelium* is poorly known and often underestimated in Nigeria and little is known about the epidemiology of this fluke in the study area. The 80.5% prevalence recorded for* Dicrocoelium* suggests the abundance of the potential primary and secondary intermediate hosts, terrestrial snail, and ants in the study area. A total of 21% of the sampled animals had mixed infection with* Fasciola* and* Dicrocoelium*; a similarly mixed infection had been reported by Ulayi et al., 2007; Ieren et al., 2016; and Magaji et al., 2016, in some parts of the country. The prevalence of mixed infection by these flukes in the present study is comparably low to the 50% reported by Ieren et al., 2016, but higher than 3.3% that was recorded by Nwosu and Srivastava (1993) and the 0.86% reported by Ulayi et al. (2007).* Fasciola* and* Dicrocoelium* are known to coexist in cattle especially in environments where the climate and ecology of the area favourably support the development and survival of their prospective intermediate hosts. Poor management often exposes the animals to the risk of acquiring these infections.

In conclusion, the present survey establishes that liver fluke infections are common among cattle in Wukari Southern Taraba State Nigeria with an association between the infection and the body condition score of the slaughtered animals. This implies that fasciolosis and dicrocoeliosis remain a major problem that can hinder the growth of the cattle production in this area. Consequently, there is a need to institute adequate control programmes in any production system involving cattle in the area. This should be achieved since animals are grazing communal land and fields along the river plains. It is important to achieve a synchronized reduction in pasture contamination with eggs of liver fluke by strategic prophylactic anthelmintic medication in all herds in Wukari complemented with well-planned management practices.

## Figures and Tables

**Table 1:** Sex, age, and body condition score prevalence of liver fluke eggs in cattle slaughtered in Wukari Abattoir, Taraba State.

Variable	Number of Samples (n=262)	Number Positive (%)		*χ* ^2^	P-value
		*Fasciola* egg	*Dicrocoelium* egg		
*Sex *					
Male	24	6 (25)	20 (83.3)	0.014	0.907
Female	238	68 (28.6)	191 (80.3)		
*Age*					
< 3 years	48	10 (20.8)	37 (77.1)	0.366	0.850
≥ 3 years	214	64 (29.9)	174 (81.3)		
*Body condition score*					
Poor	42	16 (38.1)	36 (85.7)	4.346	0.037
Average	213	58 (26.8)	169 (79.3)		
Good	7	1 (14.3)	6 (85.7)		

*Total*	262	74 (28.2)	211 (80.5)		

**Table 2:** Prevalence of mixed liver flukes egg infection in cattle slaughtered in Wukari Abattoir, Taraba State.

Type of Infection	Parasite egg	No. Positive	Prevalence (%)	*χ* ^2^	P-value
Single infection	*Dicrocoelium*	155	59.2	7.610	0.022
	*Fasciola*	18	6.9		
Mixed infection	*Dicrocoelium + Fasciola*	56	21.4		

## Data Availability

The data used to support the findings of this study are included within the article.
